# Supplementary material for: Chemotherapy-Induced Upregulation of Somatostatin Receptor-2 Increases the Uptake and Efficacy of 177Lu-DOTA-Octreotate in Neuroendocrine Tumor Cells
Source: Cancers (Basel). 2021 Jan 10;13(2):232. doi: 10.3390/cancers13020232 (PMC7828052; doi:10.3390/cancers13020232)
Supplement: Supplementary file 1 [file cancers-13-00232-s001.pdf]

Article

Chemotherapy-Induced Upregulation of Somatostatin Receptor-2 Increases the Uptake and Efficacy of ^{177}Lu-DOTA-octreotate in Neuroendocrine Tumor Cells

Rashmi G. Shah, Marine A. Merlin, Samuel Adant, Fayçal Zine-Eddine, Jean-Mathieu and Girish M. Shah

Supplementary Figs: Shah, RG: "Chemotherapy-induced …cells." (Manuscript ID: 1036587)

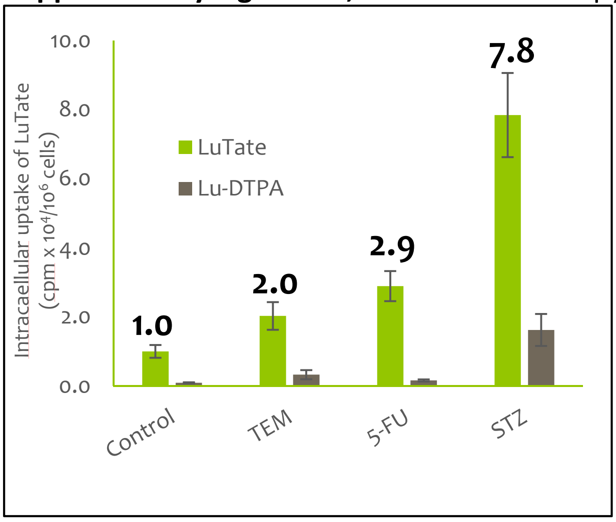

Figure S1. Increased uptake of LuTate in drug-treated cells was specific to SSTR2 binding and not due to non-specific adsorption to cells. BON-1 cells were treated for 24 h with 10 µM TEM, 10 µM 5-FU or 50 µg/mL STZ and uptake of ^{177}LuTate or ^{177}Lu-DTPA was measured after 4 days, as described for Fig. 5A. The uptake of ^{177}LuTate was increased 2 to 8-fold with different drugs, and this was between 5 to 20-times stronger than the non-specific adsorption of membrane impermeable ^{177}Lu-DTPA.

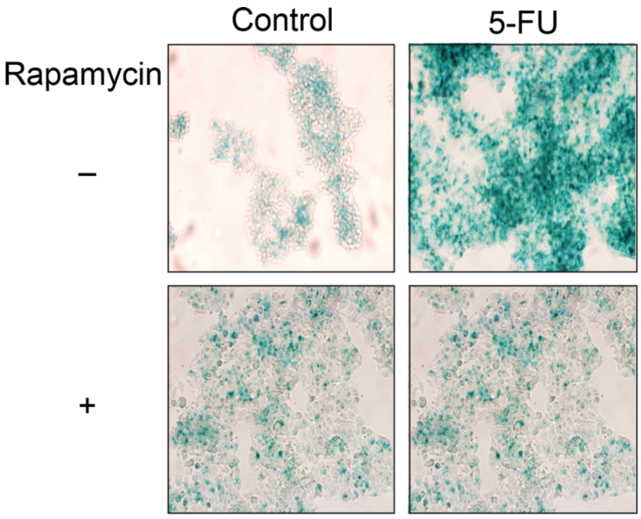

Figure 2. Senescence induced by 5-FU can be blocked by mTOR inhibitor rapamycin. BON-1 cells were treated with 50 nM of Rapamycin (or mock treated) 5 h before treatment with 5 µM 5-FU. Cells were washed at 24h and allowed to recover in fresh medium for 4 days prior to staining for the senescence-associated ß-galactosidase. .
